# Genome-scale identification of nucleosome organization by using 1000 porcine oocytes at different developmental stages

**DOI:** 10.1371/journal.pone.0174225

**Published:** 2017-03-23

**Authors:** Chenyu Tao, Juan Li, Baobao Chen, Daming Chi, Yaqiong Zeng, Honglin Liu

**Affiliations:** College of Animal Science and Technology, Nanjing Agricultural University, Nanjing, China; Qingdao Agricultural University, CHINA

## Abstract

The nucleosome is the basic structural unit of chromosomes, and its occupancy and distribution in promoters are crucial for the regulation of gene expression. During the growth process of porcine oocytes, the “growing” oocytes (SF) have a much higher transcriptional activity than the “fully grown” oocytes (BF). However, the chromosome status of the two kinds of oocytes remains poorly understood. In this study, we profiled the nucleosome distributions of SF and BF with as few as 1000 oocytes. By comparing the altered regions, we found that SF tended toward nucleosome loss and more open chromosome architecture than BF did. BF had decreased nucleosome occupancy in the coding region and increased nucleosome occupancy in the promoter compared to SF. The nucleosome occupancy of SF was higher than that of BF in the GC-poor regions, but lower than that of BF in the GC-rich regions. The nucleosome distribution around the transcriptional start site (TSS) of all the genes of the two samples was basically the same, but the nucleosome occupancy around the TSS of SF was lower than that of BF. GO functional annotation of genes with different nucleosome occupancy in promoter showed the genes were mainly involved in cell, cellular process, and metabolic process biological process. The results of this study revealed the dynamic reorganization of porcine oocytes in different developmental stages and the critical role of nucleosome arrangement during the oocyte growth process.

## Introduction

Follicles of mammals are generally categorized into preantral follicles (prior to the accumulation of antral fluid), and antral follicles (after the accumulation of antral fluid) [1–6]. This is a universally applied classification system used by the majority of researchers [7–10], but some important information such as the diameter of oocytes and the number of supporting granulosa cells in each stage are not evaluated in this system [11–12]. Torben [13] established a useful evaluation system of mouse oocytes and follicles according to the following three parameters: 1) the size of oocytes in follicles in different developmental stages, 2) the size of follicles defined by the number of granulosa cells, and 3) the morphology of the follicles. According to this system, there are three types of oocytes. An oocyte with a diameter of less than 20 μm is called a small oocyte. The growing oocyte is a cell which has begun to grow but has not reached its final size; usually, the diameter of a growing oocyte is between 20 μm and 70 μm. This is the stage with active mRNA transcription and high accumulation of energy sources. The large oocyte, also called the fully grown oocyte, is a cell that has almost reached its final size and is approximately 70 μm in diameter. Jeanine [14] had systematically performed statistical analysis of diameters of oocytes, follicles, and granulosa cells in mice, hamsters, pigs, and humans at all stages of maturation. According to the statistics, the diameter of pig oocytes in the primordial, primary, preantral, incipient antral, early antral, and Graafian follicular stages ranges from 20 to 105 μm.

Nucleosomes are the basic structural units of chromatin formed by DNA and histones [15]. Each nucleosome is composed of a 147-base pair (bp) DNA fragment that was wrapped twice around the histone octamers. The nucleosome core particles are separated by a 60-bp linker DNA [16, 17]. The nucleosomes cover most genomic DNA, except for some specific functional regions, such as promoters and enhancers that are relatively low in nucleosomes [18]. Recent studies documented that the nucleosome occupancy in promoters and the arrangement of nucleosomes around TSS is important in the regulation of gene expression [19–22]. The canonical nucleosome architecture around TSS consists of a -1 nucleosome (the first nucleosome upstream of TSS), a nucleosome depletion region (NDR), and +1, +2, +3 (and so on) nucleosomes (the first, second, and third nucleosomes downstream of TSS), which are required for gene expression [23–26]. Because the study of nucleosomes is an important branch of epigenetics, many researchers tend to study biological process from the perspective of nucleosomes. Druliner [27] profiled the nucleosome distribution in primary human lung and colon adenocarcinoma tissue, and confirmed that nucleosome reorganization is an early, widespread event and that the altered nucleosome architecture is consistent between the two samples, indicating that nucleosomes may serve as important early adenocarcinoma markers.

In our study, the growing oocytes (which are in the stage of active transcription and accumulation of mRNA and proteins) were collected with the diameter of approximately 20–30 μm from the small follicles (SF) which were less than 50 μm, while the fully grown oocytes (which have completed the stage of gene expression and accumulation, and have reached their final size) were collected from the 5–8 mm big follicles (BF). Then, we used the MNase-seq technique to generate genome-wide maps of nucleosome organization of the two samples. Our data indicated that this strategy is an efficient method for depicting the genome-wide maps of nucleosome positions with as few as 1000 cells. We found that the chromosomes of SF were more open than those of BF, and canonical nucleosome arrangements around TSS were detected in both SF and BF. However, the nucleosome occupancy of SF was less than that in BF. Our results provide insight into the regulation of nucleosome reorganization and a basis for studying the oocyte growth process.

## Materials and methods

### Collection of oocytes at different stages

All experiments were performed in accordance with the guide for the Care and Use of Laboratory Animals prepared by the Institutional Animal Care and Use Committee of Nanjing Agricultural University, China. Ovaries from 7–8-month-old commercial pigs were collected in Chengqiao slaughterhouse (Bianzhong Road NO. 78, Nanjing, China), and were transported to our lab in 1 h by a thermo with 37°C normal saline (with streptomycin and penicillin). Then, the ovaries were washed with Dulbecco's phosphate-buffered saline (DPBS, Sigma, USA) for further analysis. Follicular fluid from 5–8-mm follicles was aspirated using an 18-gauge needle attached to a 10-mL disposable syringe to retrieve the fully grown oocytes from cumulus-oocyte complexes (COCs). The COCs with multiple layers of intact cumulus cells and uniform ooplasm were selected by vacuum suction based on their morphological characteristics. The COCs were then washed twice with the wash buffer (TCM-199, 0.3% heparin, 5% amphotericin, and 10% cattle serum). The 1mg/mL hyaluronidase was used to remove the granulosa cells. The COCs were moved to about 1 mL hyaluronidase solution by vacuum suction and were repeatedly pipetted by the pipette for more than 200 times. The nude oocytes without granulosa cells were moved to the 0.5% pronase E drop for about 1 min to remove the zona pellucida. Each step was followed by washing twice in wash buffer.

To obtain the growing oocytes, the ovaries were moved to DPBS with 3 mg/mL bovine serum albumin (BSA) after washing, and cut into small pieces with a blade. Thus, the follicular fluid from follicles of different sizes and the primary oocytes went into the DPBS with BSA. The oocytes with diameters of approximately 20–30 μm were selected by vacuum suction. The growing oocytes were then washed twice in wash buffer.

### MNase-seq

A total of 1,000 large oocytes and 1,000 small oocytes were treated with EN Nucleosome DNA Prep Kit (ZYMO) according to the manufacturer’s instructions. The mono-nucleosome fragments were derived after treatment with the kit, and the library for sequencing was prepared after following the NEB protocol. The chemicals were purchased from New England Biolabs Inc. (Ipswich, MA, USA).

#### End prep

End prep was performed in a total volume of 65 μL system consisted of 3 μL end prep enzyme mix, 5 μL end repair reaction buffer (10X), and 55.5 μL nucleosome fragments, which were mixed in a sterile nuclease-free tube. The reaction was carried out at 20°C for 30 min and 65°C for 30 min.

#### Adaptor ligation

Then, 15 μL Blunt/TA ligase master mix, 1 μL ligation enhancer, and 2.5 μL adaptor were added to the last solution. The solution was incubated at 20°C for 15 min, and then 3 μL USER enzyme was added at 37°C for 15 min.

#### Cleanup of adaptor-ligated DNA

AMPure XP beads (Beckman Coulter, Inc. #A63881) were used for the cleanup of adaptor-ligated DNA fragments according to the manufacturer’s instructions.

#### PCR enrichment of adaptor-ligated DNA

PCR was performed in a 50-μL mixture containing 15 μL adaptor-ligated DNA fragments, 25 μL Q5 hot start HiFi PCR master mix, 5 μL index primer, and 5 μL universal primer. The reaction started with an initial denaturation at 98°C for 30 s, followed by 12 cycles of denaturation at 98°C for 10 s, extension at 65°C for 75 s, and a final extension at 65°C for 5 min.

#### Cleanup of PCR amplification

AMPure XP beads were used for the cleanup as mentioned earlier. After the sequencing library was prepared, it was tested with Agilent 2100. All libraries of 100-bp paired-end reads were sequenced using the Illumina HiSeq2000 system and were performed by Novogene (Beijing, China).

### Statistical analysis of data

#### Genome-wide comparison of nucleosome occupancy

The sequence reads were aligned with *Sus scrofa* (pig) reference genome *Sscrofa 10*.*2* by Bowtie2, and all uniquely matching reads were retained. A pair reads was treated as a fragment. The fragments were counted and then normalized as fragments (number of fragments in special region) per kilobase per million (FPKM) to calculate the nucleosome occupancy level [28, 29]. For each chromosome, nucleosome fragments were binned in 10-kb intervals. Comparisons between samples were conducted bin-by-bin for each chromosome. Different colors were used to represent the level of change of nucleosome occupancy between the two samples; red indicated a ≥1.5-fold increase in SF, green indicated a ≥1.5-fold decrease in SF, grey indicated the absence of detected nucleosomes, and yellow indicated all other cases. The same approach was used in the nucleosome occupancy comparison, and the whole genome was scanned by a 500-bp window. The FPKM value was calculated in each window and compared pairwise between samples for the differential analysis. The window in which the FPKM value was upregulated or downregulated two-fold was selected.

#### Nucleosome occupancy in different regions of genome

Nucleosome distribution across the genome was further explored by calculating the percentage of nucleosome reads in more detailed regions (i.e., promoter, 5’ UTR, exon, intron, 3’ UTR, intergenic regions). The specific sequence information of these regions corresponding to the reference genome *Sscrofa 10*.*2* was downloaded from UCSC. The nucleosome occupancy in each region was calculated using BEDTools software (version 2.16.2)^37^, and the nucleosome occupancy ratio in each region was counted. The results were compared with the genome ratio of the different regions. A 500-bp window was used to scan the genome. According to the GC content, the 500-bp fragments were divided into five portions, the FPKM value for all the windows was calculated, and the relationship was analyzed between GC content and nucleosome occupancy.

#### Nucleosome distribution profiles

The gene annotation file was downloaded from University of California at Santa Cruz (UCSC) Genome Bioinformatics. Nucleosomes within 1 kb of the TSS were collected. The total 2-kb length was binned in 10-bp intervals, and the RPKM value in each bin was calculated to get a profile of nucleosome distribution around the TSS [29].

#### GO analysis

We used a 500-bp window to scan the genome, and found the windows in which the nucleosome occupancy changed two-fold (upregulated two-fold in SF or downregulated two-fold in SF) between SF and BF. The differential windows were aligned to the genome, and the corresponding gene was selected if the 500-bp fragment fell in a genic region. To find the differential genes more accurately, the same method was used except that we selected the genes in which the promoter region had a two-fold change in nucleosome occupancy. After picking out the genes, we carried out the gene function enrichment analysis using the online analysis software WEGO (http://wego.genomics.org.cn/cgi-bin/wego/index.pl) [30] developed by BIG, China.

## Results

### Growing oocytes and fully grown oocytes collection

**Fig 1:**
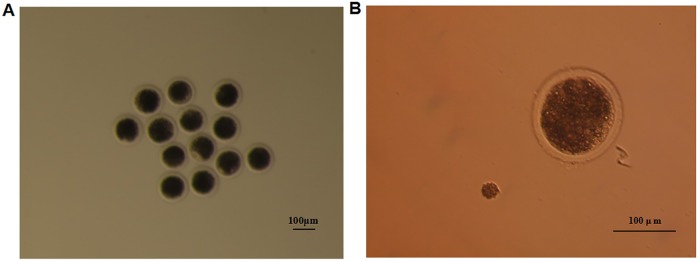
Collection of the porcine oocytes of different growing stages. **(A)** Collection of the porcine “large” oocytes. **(B)** Comparison of the “large” and “growing” oocytes.

After being selected under the stereoscope, the COCs were treated with hyaluronidase to obtain the nude large oocytes (Fig 1A). The diameter of the large oocytes was approximately 120 μm, while the diameter of the growing oocytes was approximately 20–30 μm (Fig 1B). There were lipid droplets in both types of oocytes, which was a salient feature of the oocytes. An obvious volume increase in the two kinds of oocytes was observed, suggesting that an accumulation of cytoplasm took place during the process of oocyte growth.

### Successful establishment of 1000 cell MNase-seq

**Fig 2:**
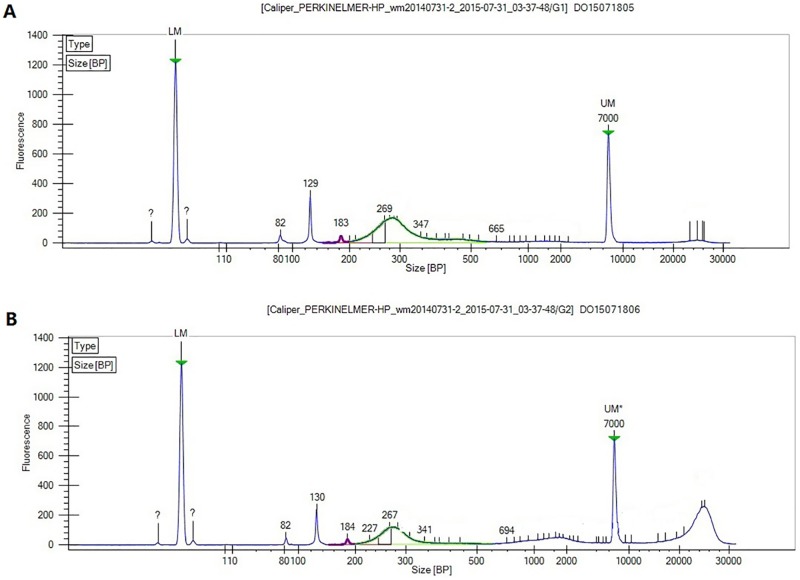
Agilent 2100 detection of adaptor-ligated mononucleosome library of “large” oocytes (A) and “growing” oocytes (B).

In our study, approximately 1000 large and growing porcine oocytes were derived and the cells were digested by MNase to obtain the mononucleosome fragments, followed by the construction of sequencing for next-generation sequencing (see details in Methods). Fig 2A and 2B showed the Agilent 2100 detection results of the adaptor-ligated mononucleosome. The main peaks of the library are 269 bp and 267 bp, which consist of the indexed adaptor (which was 120 bp) and the mononucleosome fragment (which is approximately 150 bp). The standard architecture of the nucleosome is 147 bp. However, in the genome there exist loose and tight types that may have a length of more or less than 147 bp. Furthermore, the fragments derived from enzyme digestion are not of the same length; thus, in the Agilent detection results, the main peak spanned from 230 bp to 300 bp. This fact validated that this method was effective in generating a mononucleosome sequencing library.

### Global nucleosome occupancy is reorganized during oocyte growth

Sequencing was conducted on an Illumina HiSeq^™^ 2000 platform, and a total of 95 million and 107 million reads were obtained of BF and SF. The raw sequenced data had been submitted to the sequence read archive (SRA) database, the accession number was PRJNA347494. The details of the sequencing data and mapping information are shown in Supplementary S1 and S2 Tables, respectively. Sequencing data showed the effectiveness of the MNase-seq system. The detected nucleosome coverage rates of BF and SF were 72% and 77%, respectively (Supplementary S2 Table), which indicates that much of the genomic DNA forms the structure of the nucleosome.

**Fig 3:**
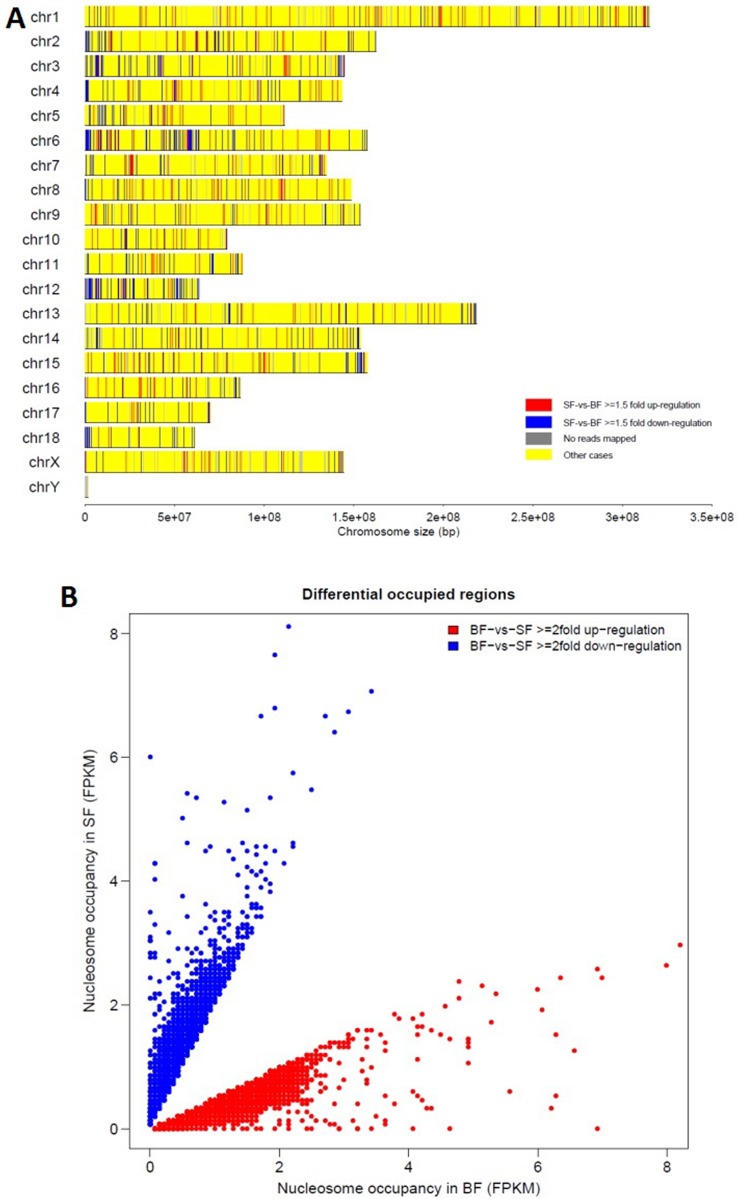
Nucleosome occupancy comparison between SF and BF. **(A)** Genome-wide comparison of nucleosome occupancy of SF and BF. Colors indicate the change in nucleosome occupancy in each 10 kb region between the two samples. Red indicates a ≥ 1.5-fold increase in nucleosome occupancy of SF, green indicates a ≥ 1.5-fold decrease in nucleosome occupancy of SF, grey indicates the no reads mapped region, and yellow indicates regions with other cases. **(B)** Differential analysis of nucleosome occupancy in SF and BF. One dot represents a window of which the nucleosome occupancy difference between the two samples is more than 2 folds. Colors indicate the change in nucleosome occupancy in each 500bp window between the two samples. Red indicates a ≥ 2-fold increase in nucleosome occupancy of BF, blue indicates a ≥ 2-fold decrease in nucleosome occupancy of SF.

To investigate how the chromosome is dynamically changed during the oocyte growth process, we used 10 kb and 500 bp respectively to scan the genome to find the windows with different nucleosome occupancies and how they distributed across the genome. In Fig 3A, red indicated the nucleosome increase in SF, and blue indicated the nucleosome loss in SF. Viewed as a whole, chromosomes all showed nucleosome change, and the blue windows were much more than the red ones; thus, the chromosome status tended toward nucleosome loss and a more open configuration in SF, but a relative tight configuration in BF (Fig 3A). Far stricter standards of a 500 bp window and a two-fold change were used in the next analysis. The same result was found, with 10,963 windows showing nucleosome decrease in SF (red in Fig 3B) and 7776 windows (blue in Fig 3B) showing nucleosome increase in SF. The two results validated that the chromosomes of SF were more open than those of BF, suggesting higher transcriptional activity in SF.

### Nucleosome occupancy changes in different regions of the genome

We classified the genome into the two different functional regions of genic and intergenic. The genic region was further divided into the promoter, 5’ untranslated regions (UTRs), exons, introns, and 3’ UTRs, and the FPKM value of the nucleosome occupancy in each region was calculated (Table 1). The first column of the table is the ratio of each region to the whole genome, and the second and third columns show the nucleosome ratio of BF and SF, respectively. The nucleosome occupancy decreased in the intergenic region and increased in the genic region more than it should have. From SF to BF, the nucleosome occupancy in the intergenic region increased from 68.79% to 70.46%, and the promoter region also increased in nucleosome occupancy, suggesting higher transcriptional activity in SF. The nucleosome occupancy of the coding region decreased in BF than SF.

**Table 1:** Nucleosome ratios of BF, SF in different functional elements.

Genomic items	Genome Ratio (%)	Nucleosome Ratio of BF (%)	Nucleosome Ratio of SF (%)
All	100	100	100
Promoter	1.07	1.5	1.38
5’ UTR	0.1	0.18	0.19
Exon	1.94	2.82	2.79
Intron	22.95	24.57	26.13
3’UTR	0.49	0.59	0.59
Intergenic	73.44	70.46	68.79

**Fig 4:**
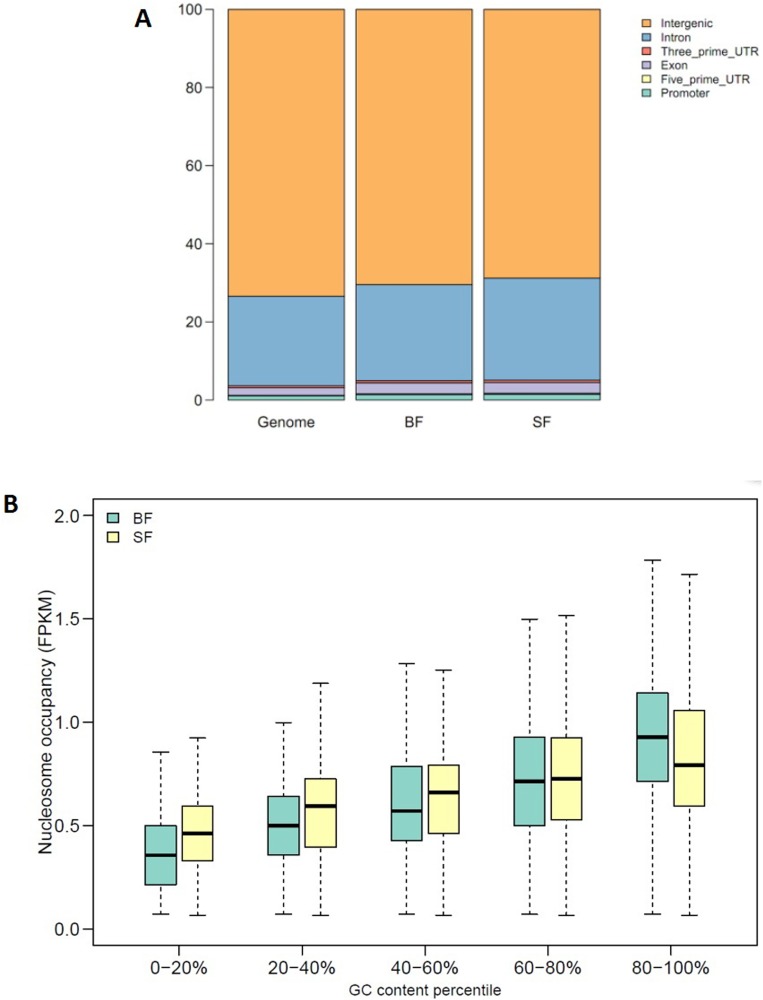
Global nucleosome occupancy of SF and BF. **(A)** Ratio of different functional elements in whole genome, SF, and BF. The genome bar indicates the percentage of all the functional elements in porcine genome, while SF and BF bars indicate the nucleosome occupancy ratio of every functional element in the two samples. **(B)** Boxplot showing the relationship between nucleosome occupancy and GC content. The blue box indicates BF and the yellow box indicates SF.

Next, we explored the relationship between nucleosome occupancy and the GC content, and found that there was a positive correlation between them. The nucleosome occupancy increased with increasing GC content (Fig 4B). Interestingly, we found that in regions with low GC content, the nucleosome occupancy of SF was higher than that in BF, while in regions with high GC content, the nucleosome occupancy of SF was lower than that in BF, suggesting more open chromosome architecture in high GC content regions of SF.

### Nucleosome occupancy around TSS

**Fig 5:**
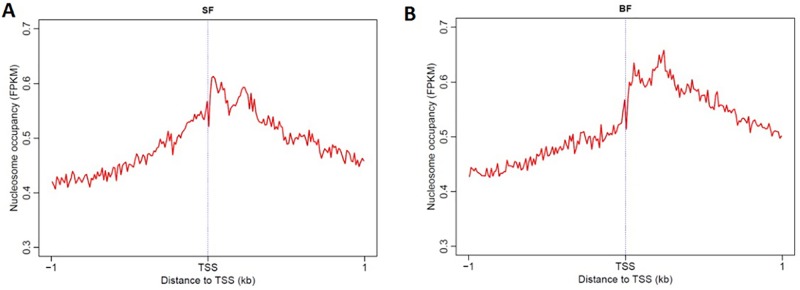
Nucleosome arrangement around TSS (A) and in the genic region (B) of all the genes in SF and BF.

Previous studies have validated that the nucleosome occupancy at the promoter region is related to the gene expression level. Less nucleosomes in the promoter leads to higher transcriptional activity, while more nucleosomes in the promoter leads to low expression levels. Additionally, the arrangement of nucleosomes around the TSS is crucial for gene expression. The positioning of -1, NDR, +1, +2, etc. of nucleosomes around the TSS is canonical in highly expressed genes. In our research, we calculated the nucleosome occupancy and arrangement around the TSS of all the genes in SF and BF, and found that both showed canonical nucleosome distributions around the TSS (Fig 5A and 5B). The FPKM value of +1 and +2 nucleosomes in SF was approximately 0.6, while the value was higher than 0.65 in BF. In SF, the overall nucleosome occupancy around the TSS was higher than that in BF.

### GO analysis of genes with different nucleosome occupancy

**Fig 6:**
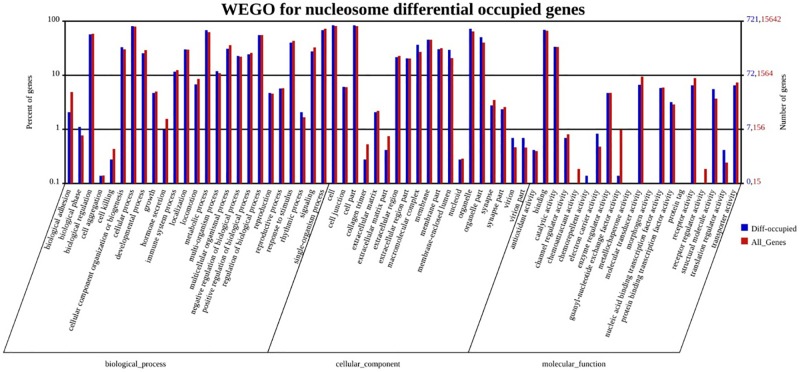
GO functional annotation of genes with different nucleosome occupancy in promoter. Horizontal axis shows the three categories and the detailed GO terms, while ordinate shows numbers (right) and the percentages (left) of all the genes (red) and the genes with different nucleosome occupancy in promoter (blue).

First, we selected the genes with different nucleosome occupancy in the genic region (consisting of promoter, 5’UTR, exon, inton, 3’UTR). A total of 2550 genes were selected. GO analysis of the 2550 genes showed that the genes were involved mostly in the biological processes of cell, cell part, binding and so on. Because the nucleosome occupancy in the promoter plays an important role in gene expression regulation, we then selected the genes with different nucleosome occupancy in the promoter, after which a total of 721 genes remained. The specific genes are listed in S2 Table. The nucleosome occupancies in the promoter region of these genes were two-fold changed including both two-fold increase and decrease. According to the results of GO analysis of the 721 genes (Fig 6), the top five biological processes involving the genes were cell, organelle, cellular process, metabolic process, and cell part. From the GO analysis, it is evident that the results of gene function enrichment analysis showed the same trend for all the genes of porcine; this may be because SF had overall higher transcriptional activity than BF.

## Discussion

The oocyte growth process is a complex biological process during which there is an accumulation of lipid, proteins, and other energy-providing macromolecules, which leads to an increase in the volume of the oocyte. The growing oocytes show high transcriptional activity, while the fully grown oocytes show low transcriptional activity [31–37]. Therefore, we suspect that there may be a big difference between the chromosome status of the growing and fully grown oocytes, but little research has been carried out to clarify this. Thus, we chose porcine oocytes in two developmental stages and attempted to compare their differences in nucleosome occupancy and distribution. In this study, because the experimental materials are precious and it is difficult to obtain large quantities of porcine oocytes, a total of 1000 oocytes in different stages were chosen. The method of establishing an MNase-seq library with as few as 1000 cells was used successfully.

From growing oocytes to fully grown oocytes, the nucleosome across the genome is depleted as a whole, suggesting that the chromosome status of growing oocytes is more open, which may be the reason for higher transcriptional activity. The coverage rate in SF is higher than that in BF, and we speculated that this may be because of an error in constructing libraries and in the sequencing process. The results of nucleosome distribution analysis in different functional regions revealed depleted nucleosome occupancy in the promoter of SF, and an increase in genic regions (regions except for intergenic region) in SF. This may be a marker of higher gene expression. The nucleosome occupancy of SF is higher than that in BF in the low GC content region, while in the high GC content region, the nucleosome occupancy is lesser in SF than that in BF. As reported in a previous study, GC content has a positive correlation with gene density [38–40]; that is, the GC content of genic region is normally higher than that in other regions of the genome. In the genic region, the promoter is normally GC-rich. Thus, the results of nucleosome depletion in high GC content regions in SF coincides with the nucleosome occupancy distribution results in the genome, and this may lead to higher transcriptional activity.

Analysis of the nucleosome distribution around the TSS of growing oocytes and fully grown oocytes showed basically same both with NDR, +1, +2 etc. nucleosome. According to our assumption, the growing oocytes with high transcriptional activity may have the canonical nucleosome arrangement around the TSS with NDR, +1, +2, etc. nucleosomes, but the nucleosome arrangement around the TSS of fully grown oocytes may not be canonical. However, the results also show obvious NDR, +1, +2, etc. nucleosomes in fully grown oocytes. This may be because we selected all the genes of porcine in this analysis, and even though the fully grown oocytes have low transcriptional activity, there are still many genes expressed to maintain a normal cell’s morphology and existence (e.g., the housekeeping genes). Therefore, the nucleosome distributions of the two kinds of oocytes are the same. However, the nucleosome occupancy in the promoter and coding region (from TSS to TES) of growing oocytes is lower than that in fully grown oocytes, which indicates a higher gene expression level; this is consistent with our assumption.

Because we know that the nucleosome occupancy in the genic region is a significant factor in gene expression regulation [41–42], 2550 genes in which the differential fragments of two-fold change in nucleosome occupancy are aligned to were selected. Furthermore, we selected 721 genes with differential nucleosome occupancy in the promoter, including two-fold increase and decrease, and GO analysis of the two groups’ genes showed different functional enrichment results. Since we didn’t carry out the RNA-seq of pig “fully-grown” oocytes and “growing” oocytes, we cannot get the exact relationship between the nucleosome changed genes and the tanscriptome changes. However, according to the already reported study, the function of nucleosomes in regulating gene expression is mostly determined by assessing the occupancy and arrangement of nucleosomes in the promoter [25, 26]. Thus we only analyzed the genes with different nucleosome occupancy in the promoter, hoping to find some critical genes in regulating oocytes growing. From the above, we believe that the second result is more accurate for the purpose of studying the critical genes involved in the oocyte growth process. The top biological processes are cell, organelle, cellular process, and metabolic process, and these processes all have a relationship with cell development and growth. Thus, we believe that the results of our study reveal important changes occurring in chromosomes and provide insight into critical candidate genes in porcine oocyte growth.

## Supporting information

S1 Table Basic information of the MNase-seq data from BF and SF. (XLS)

S2 Table Mapping information of the data from BF and SF. (XLS)

S3 Table GO analysis of genes with different nucleosome occupancy in promoter. (XLS)
